# A Protein-Based, Long-Acting HIV-1 Fusion Inhibitor with an Improved Pharmacokinetic Profile

**DOI:** 10.3390/ph15040424

**Published:** 2022-03-30

**Authors:** Wei Xu, Zhe Cong, Qianyu Duan, Qian Wang, Shan Su, Rui Wang, Lu Lu, Jing Xue, Shibo Jiang

**Affiliations:** 1 Shanghai Public Health Clinical Center, Key Laboratory of Medical Molecular Virology (MOE/NHC/CAMS), School of Basic Medical Sciences, Shanghai Institute of Infectious Disease and Biosecurity, Fudan University, Shanghai 200032, China; xuwei11@fudan.edu.cn (W.X.); 19111010059@fudan.edu.cn (Q.D.); wang_qian@fudan.edu.cn (Q.W.); 09301010057@fudan.edu.cn (S.S.); 2 NHC Key Laboratory of Human Disease Comparative Medicine, Beijing Key Laboratory for Animal Models of Emerging and Remerging Infectious Diseases, Institute of Laboratory Animal Science, Chinese Academy of Medical Sciences and Comparative Medicine Center, Peking Union Medical College, Beijing 100021, China; congz@cnilas.org; 3 Beijing Prosperous Biopharm Company, Beijing 100021, China; wangr@prospbiopharm.cn

**Keywords:** HIV-1, gp41, fusion inhibitor, human serum albumin, long-acting inhibitor

## Abstract

Recently, a series of highly effective peptide- or protein-based HIV fusion inhibitors have been identified. However, due to their short half-life, their clinical application is limited. Therefore, the development of long-acting HIV fusion inhibitors is urgently needed. Here, we designed and constructed a protein-based, long-acting HIV fusion inhibitor, termed FLT (FN3-L35-T1144), consisting of a monobody, FN3, which contains an albumin-binding domain (ABD), a 35-mer linker (L35), and a peptide-based HIV fusion inhibitor, T1144. We found that FLT bound, via its FN3 component, with human serum albumin (HSA) in a reversible manner, thus maintaining the high efficiency of T1144 against infection by both HIV-1 IIIB (X4) and Bal (R5) strains with IC_50_ of 11.6 nM and 15.3 nM, respectively, and remarkably prolonging the half-life of T1144 (~27 h in SD rats). This approach affords protein-based HIV fusion inhibitors with much longer half-life compared to enfuvirtide, a peptide-based HIV fusion inhibitor approved for use in clinics. Therefore, FLT is a promising candidate as a new protein-based anti-HIV drug with an improved pharmacokinetic profile.

## 1. Introduction

HIV/AIDS has continuously posed a threat to global public health [1,2]. As of 2021, about 36.3 million people have died of AIDS worldwide, and 37.7 million people are still living with HIV/AIDS [3,4].

Currently, 24 HIV therapeutic drugs have been approved by the US FDA, including 5 nucleoside reverse transcriptase inhibitors (NRTIs), 5 non-nucleoside reverse transcriptase inhibitors (NNRTIs), 6 protease inhibitors (PIs), 3 integrase strand transfer inhibitors (INSTIs), one fusion inhibitor, one CCR5 antagonist, one attachment inhibitor, one post-attachment inhibitor and one pharmacokinetic enhancer. In addition, 23 combination HIV medicines are available, each one including two or more HIV drugs [5,6,7]. 

**Figure 1:**
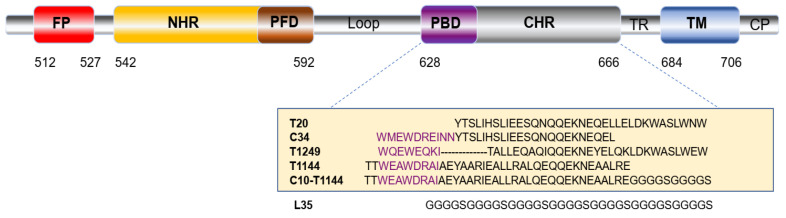
Schematic view of the HIV-1HXB2 gp41 molecule and peptide sequences of the CHR-derived peptides. The gp41 consists of fusion peptide (FP), N-terminal heptad repeat (NHR), pocket-forming domain (PFD), pocket-binding domain (PBD), C-terminal heptad repeat (CHR), transmembrane domain (TM) and cytoplasmic domain (CP). The PBD sequence in the CHR-derived peptides is highlighted in purple. The amino acid sequences of T20, C34, T1249, T1144 and C10-T1144 peptides as well as the linker, L35, are shown. The T20, C34, T1249, T1144, and C10-T1144 peptides, which are fully or partially derived from the HIV-1 gp41 CHR domain, can bind the viral NHR to prevent the formation of the homologous 6-HB between the viral NHR and CHR regions.

T20 (generic name: enfuvirtide; brand name: Fuzeon), a 36-amino-acid peptide derived from the natural sequence (aa 643–678) of the HIV-1 gp41 C-terminal heptad repeat (CHR) domain (Figure 1), is the first US FDA-approved HIV fusion inhibitor for clinical use to treat HIV-infected patients who have failed to respond to antiretroviral drugs (Figure 1) [8]. However, its short half-life (about 3.46 h to 4.35 h) in humans [9] and low potency have limited its clinical application. Therefore, it is essential to design and develop new HIV fusion inhibitors with improved pharmacokinetics (PK) and potency [10,11,12]. 

Since T20 does not contain the pocket-binding domain (PBD), it cannot strongly bind to the pocket-forming domain (PFD) in the gp41 N-terminal heptad repeat (NHR) region to form stable 6-helix bundle (6-HB) [13]. Consequently, the mutations in GIV motif region (aa 36–45) in the NHR region, such as V38E and N42S, result in its low genetic barrier to resistance [14]. Tl144 is 38-amino-acid peptide with a sequence (Figure 1) modified from that of T651 peptide derived from the natural sequence (aa 626–673) of the HIV-1 gp41CHR domain [15]. Unlike T20, T1144 contains the PBD and can strongly bind to the PFD in the gp41 NHR region to form stable 6-helix bundle (6-HB) [16]. Therefore, T1144 has high genetic barrier to resistance [15] and the mutations in GIV motif region of the viral NHR region have no significant effect on T1144’s antiviral activity. Therefore, we chose T1144 for this study.

One promising strategy to extend the half-life of peptide drugs involves crosslinking the peptide to albumin [17] since human serum albumin (HSA) can stay in the blood circulation between two and four weeks [18,19]. However, we cannot allow T1144 to directly bind HSA through non-reversible interaction since the T1144-HSA complex has a molecular weight >70 kDa, while the constrained space of NHR-trimer in the fusion intermediate state only allows a molecule with a molecular weight <40 kDa to be accessed [20]. However, we can link T1144 with a small-molecule monobody, FN3, which contains an albumin-binding domain (ABD), through the long flexible linker L35. The resultant protein, FN3-L35-T1144 (hereinafter termed FLT) with a molecular weight of ~22 kDa is expected to bind to HSA through a reversible interaction. Therefore, the released FLT from HSA can easily access and interact with the NHR-trimer in the fusion intermediate state. The long linker, L35, between FLT and HSA may allow the HSA-bound FLT to access and interact with the NHR-trimer to form heterologous 6-HB and block virus-cell fusion. 

In this study, we constructed a plasmid encoding FLT, expressed and purified FLT, determined its biophysical properties, and evaluated it in vitro anti-HIV-1 activity and in vivo protection of nonhuman primates (NHPs) against simian/human immunodeficiency chimeric virus (SHIV) infection. 

## 2. Results

### 2.1. Construction and Expression of FLT

**Figure 2:**
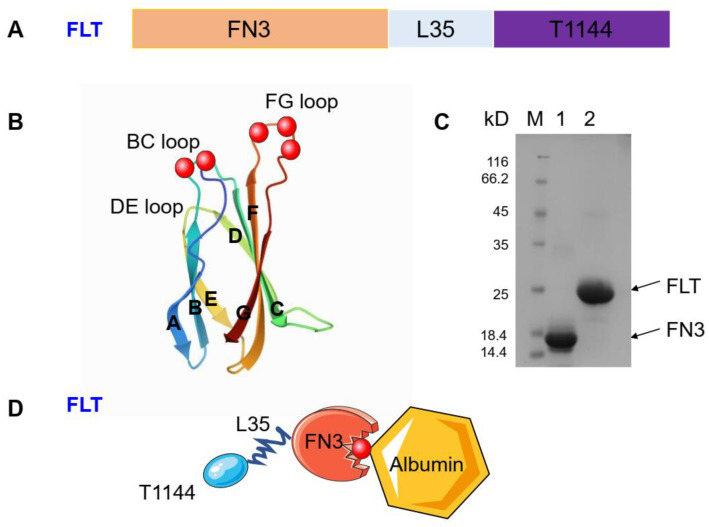
The composition of FLT, its identification with SDS-PAGE and schematic drawing of its interaction with albumin. (**A**) The composition of FLT. FLT consists of three parts, including FN3, L35 and T1144. FN3, a domain of a unique scaffold monobody, has a low molecule size and can reversibly bind with human serum albumin (HSA). (**B**) 3D visualization of FN3 (PDB: 1TTG). ABD was labeled in red. (**C**) SDS-PAGE analysis of the purified FN3 and FLT. FLT and FN3 in soluble form were abundantly expressed and released into the *E. coli* culture supernatant and purified with Ni Sepharose column. (**D**) Schematic show of a working model for FLT binding with albumin.

In order to maximize the expression of FLT, the coding region was optimized with codons commonly used in *E. coli* FN3 and FLT were expressed and purified (Figure S1). Surprisingly, even though FLT contains many hydrophobic amino acids, it was expressed up to 12.0 mg/L in soluble form in the cytoplasm of *E. coli*, possibly since FN3 does not contain disulfide bonds (Figure 2).

### 2.2. FLT Exhibited Potent Inhibitory Activity against Infection by Laboratory-Adapted HIV-1 Strains and Divergent Primary HIV-1 Isolates

**Figure 3:**
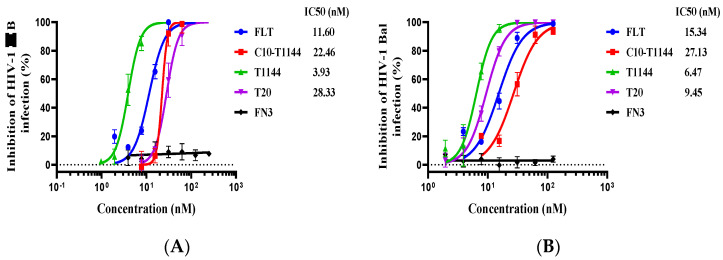
Antiviral activity of FLT against the laboratory-adapted HIV-1 stains. (**A**) Inhibition of FLT on infection by HIV-1 IIIB; (**B**) Inhibition of FLT on infection by HIV-1 Bal. Each sample was tested in triplicate and the experiment was repeated three time. The data from a representative experiment are presented as mean ± standard deviation.

Next, we studied the expression of FLT in *E. coli* for putative antiviral activity. We first tested the inhibitory activity of FLT against infection of HIV-1 IIIB (X4 tropic) strain, including FN3, T1144, T20 and C10-T1144, which contains a 10-mer linker (GGGGSGGGGS) at the N-terminus of T1144, as controls. As shown in Figure 3A, FLT inhibited HIV-1 IIIB infection in a dose-dependent manner with an IC_50_ (half maximal inhibitory concentration) of 11.6 nM, while FN3 showed no detectable inhibitory activity, and the IC_50_ of values of T1144, C10-T1144 and T20 were 3.9, 22.5, and 28.3 nM, respectively. Consistently, the IC_50_ values of FLT, T1144, C10-T1144 and T20 for inhibiting infection by HIV-1 Bal (R5 tropism) were 15.3, 6.5, 27.1, and 9.5 nM, respectively (Figure 3B). These results indicate that FLT exhibits anti-HIV-1 activity similar to that of C10-T1144, in turn suggesting that conjugation of FN3 to C35-T1144 does not significantly affect the antiviral activity of linker-linked T1144. 

Next, we assessed the inhibitory activity of FLT (FN3 and T20 as controls) on infection by a panel of clinical HIV-1 isolates with different subtypes (A, B, C, D, and AG). Strikingly, we found that FLT could effectively inhibit infection by all clinical HIV-1 isolates in this panel with IC_50_ values ranging from 6.4 to 65.3 nM, while the IC_50_ values of T20 were in the range of 21.1~77.1 nM. FN3 showed no inhibitory activity at the concentration up to 125 nM (Table 1). We compared the Env sequences of the HIV-1 isolates listed in Table 1 and found that the average identity and similarity of the 7 available NHR sequences are 90.1 and 96.3%, respectively, while those of the 10 CHR sequences are 77.7% and 91.3%, respectively (Table S1), suggesting that the relatively conserved NHR sequences may explain why the NHR-targeting fusion inhibitors, FLT and T20, have broad-spectrum antiviral activity against divergent HIV-1 isolates.

### 2.3. FLT Effectively Inhibited Infection by Drug-Resistant HIV-1 Strains

The emergence of drug-resistant HIV-1 strains is still a major issue for HIV treatment as many anti-HIV drugs induce drug-resistant strains within weeks to years after drug treatment [21]. Here we tested the inhibitory activity of FLT against infection by drug-resistant strains. First, we investigated whether FLT, which contains the T1144 part, a T20-like sequence, is effective against T20-resistant strains or not. As shown in Table 2, T20 was effective against infection by the T20-sensitive strain HIV NL4-3 with IC_50_ of about 21 nM, but not effective against infection by three T20-resistant strains (N42T/N43K, V38E/N42S and V38A/N42T) at concentration as high as 128 nM. However, FLT was effective against both T20-sensitive and -resistant strains with IC_50_ ranging from 19.4 to 28.1 nM. Next, we tested the inhibitory activity of FLT against NRTI/NNRTI-resistant strains and found that the NRTI-sensitive strain (A018A) and two resistant strains (A012 and IIIB A17) were equally sensitive to the inhibition of FLT with IC_50_ ranging from 23.2 to 28.6 nM. Similarly, FLT proved highly effective against infection by three integrase inhibitor-resistant strains with IC_50_ ranging from 20.2 to 22.3 nM (Table 2). These results suggest that FLT has potential to be developed for clinical use to treat patients who are infected by multidrug-resistant HIV-1 strains.

### 2.4. FLT Interfered with gp41 Six-Helix Bundle (6-HB) Formation

**Figure 4:**
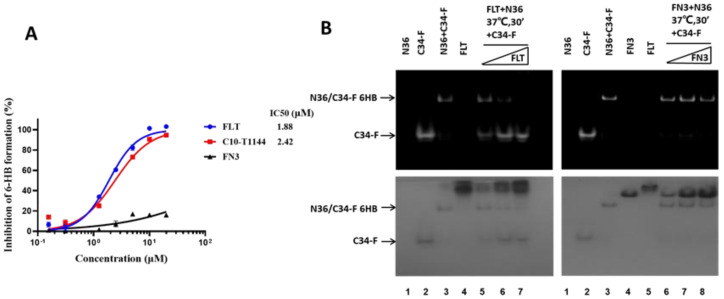
The mechanism of action of FLT. (**A**) A competition ELISA was used to detect the potential inhibitory activity of FLT, C10-T1144, and FN3 on 6-HB formation between N36 and C34 peptides. Using 6-HB-specific monoclonal antibody NC-1. For experimental details, see Section 4.6. (**B**) FN-PAGE analysis of FLT-mediated inhibition of 6-HB formation between N36-peptide and the FAM-labeled C34 peptide (C34-FAM). For experimental details, see Section 4.5.

To elucidate whether the mechanism of action of FLT that contains a T1144 part is similar to that of T1144, we tested whether FLT (control: T1144) effectively inhibited 6-HB formation between the gp41 NHR and CHR peptides with an ELISA using a 6-HB-specific monoclonal antibody (mAb), NC-1 [22]. We found that both FLT and C10-T1144 inhibited 6-HB formation in a dose-dependent manner with IC_50_ of 1.88 and 2.42 μM, respectively, while FN3 exhibited no significant inhibition at the concentration up to 10 μM (Figure 4A). In addition, we performed fluorescence native polyacrylamide gel electrophoresis (FN-PAGE) using N36 peptide and fluorescence-labeled C34 peptide (C34-F) to determine the effect of FLT on the formation of 6-HB between the N36 peptide and C34-F peptides as previously described [23]. We performed FN-PAGE by loading N36 (lane 1), C34-F (lane 2), N36 + C34-F mixture (lane 3), FLT (lane 4), and the mixtures of N36 + C34-F + FLT at 10, 20, and 40 μM (lanes 5-lane 7) to the gel, respectively. The gel was imaged with a FluorChem 8800 imaging system (Figure 4B, left and upper panel) and then stained with Coomassie Blue (Figure 4B, left and lower panel). Since N36 has a net positive charge, it migrated upward and off the gel (lane 1). However, C34-F carries a net negative charge; therefore, it migrated downward and displayed a band in the lower part of the gel (lane 2). The N36 + C34-F mixture formed a complex (6-HB) that showed a band in the upper part of the gel (lane 3), suggesting that C34-F peptide wasin the 6-HB complex. FLT showed a major band in the top position of the gel (lane 4). By increasing the concentration of FLT in the N36 + C34-F + FLT mixtures, the density of the 6-HB band was gradually decreased, while the density of the C34-F band was gradually increased (lanes 5~7). These results suggest that FLT binds to N36 and blocks the binding of C34-F to N36 to form 6-HB in a dose-dependent manner. FN3, as a control, did not interact with N36 and could not block the 6-HB formation between N36 and C34-F (Figure 4B, right panels).

### 2.5. FLT Bound to HSA as Measured by Isothermal Titration Calorimetry (ITC)

**Figure 5:**
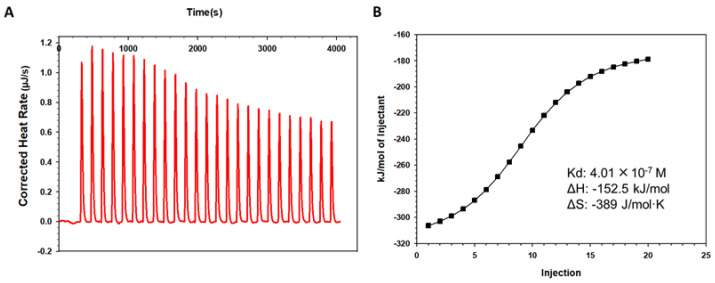
Measurement of K_d_ between FLT and the HSA by ITC. The interaction affinity was determined by low volume Nano ITC calorimeter at 25 °C. (**A**) Raw data of titration traces; (**B**) binding affinities between FLT and the HSA. FLT and HSA were dialyzed in PBS for 16 h. FLT (20 μM, 185 μL) was placed to the active cell, while HSA (380 μM, 50 μL) in the titration needle. Data acquisition and analysis were performed using TA-ITC software. The observed K_d_, ΔH and ΔS for this interaction are 4.01 × 10^−7^ M, −152.5 kJ/mol, −389 J/mol·K, respectively. The experiments were repeated twice, and the representative data are shown.

Since the binding of FLT to HSA is a reversible reaction, we performed isothermal titration calorimetry (ITC) to determine the thermodynamic parameters of FLT-HSA binding. We found that FLT bound to HSA with a binding constant (K_d_) of 4.01 × 10^−7^ M. Under 25 °C constant temperature condition, ∆H and ∆S were −152.5 kcal/mol and -389 J/mol·K, respectively, suggesting that Van der Waals and hydrophobic interaction are the main binding forces for FLT-HSA (Figure 5) [24]. Therefore, these results suggest a non-covalent binding interaction between FLT and HSA. Affinity is one of the key functional properties of drug efficacy. In many cases, it is necessary to select the appropriate kinetic parameters according to the mechanism of drug action. The average K_d_ values of the anti-HIV drugs, including the protease inhibitors (e.g., atazanavir), the non-nucleoside reverse transcriptase inhibitors (e.g., efavirenz) and the nucleoside reverse transcriptase inhibitors (e.g., abacavir) binding HSA ranged between 4.46 × 10^−5^ M and 3.8 × 10^−4^ M [25].

### 2.6. FLT Displayed Improved Pharmacokinetic Profiles in Rats

**Figure 6:**
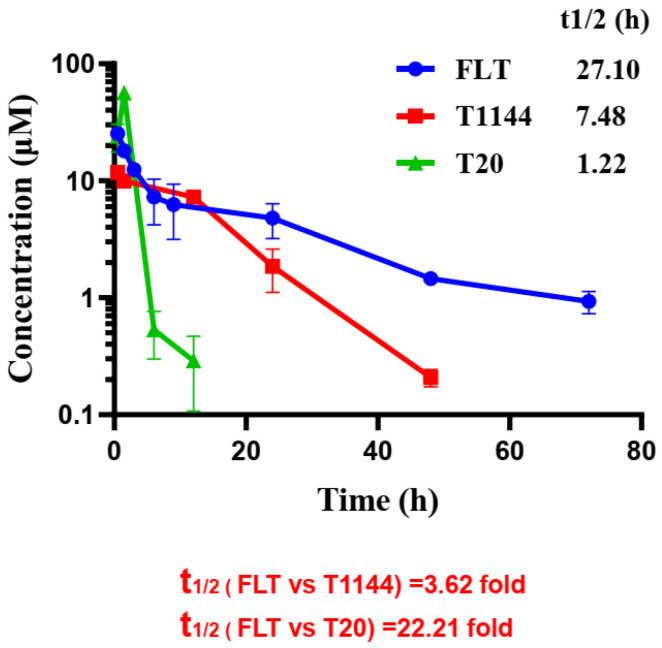
Time-course plasma concentration profiles of FLT, T1144 and T20. Single-dose of FLT (5.94 mg/kg), T1144 (1.28 mg/kg) or T20 (1.26 mg/kg) was administered intravenously in SD rats (*n* = 3). Blood samples were collected from the orbital sinus at 0, 0.5, 1.5, 3, 6, 9, 12, 24, 48, 72, 96, and 120 h after injection of the inhibitors tested, respectively, after injection of the inhibitors tested. Concentrations of FLT, T1144 and T20 were determined by ELISA. PK parameters are shown in Table 3.

To evaluate the pharmacokinetic (PK) profiles in vivo, we analyzed the PK characteristics of FLT in rats following intravenous injection. Based on plasma concentration distribution, the plasma half-life (t_1/2_) of T20, T1144 and FLT was 1.22, 7.48 and 27.10 h, respectively, indicating that FLT’s t_1/2_ is 22.2- and 3.6-fold longer than that of T20 and T1144, respectively (Figure 6). The results of non-compartmental PK analysis showed that the clearance of FLT was 0.063 mL/h/kg, much lower than that of T1144 (0.349 mL/h/kg) and T20 (0.855 mL/h/kg), respectively (Table 3).

### 2.7. FLT Exhibited In Vivo Efficacy against Chronic SHIV Infection in Nonhuman Primates (NHPs)

**Figure 7:**
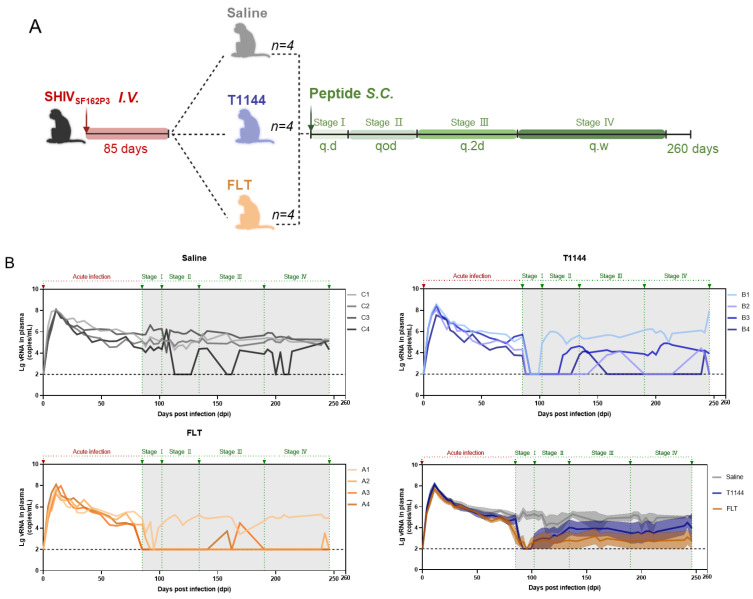
Therapeutic efficacy of FLT in chronic persistent infection of monkeys. (**A**) Schematic diagram of the therapeutic experimental design. Twelve monkeys (4 for each group) were infected with SHIV_SF162P3_ at day 0, followed by programmed subcutaneous injections as shown; (**B**) Plasma viral loads of monkeys in saline, T1144 and FLT groups and comparison of the average of the changes of plasma HIV-1 RNA in each group. The black dash line designates the limit of detection. For experimental details, see Section 4.9.

We further examined the in vivo therapeutic effect of FLT in a NHP model. First, we established a rhesus monkey model chronically infected with SHIV_SF162P3_, in which virus replication reached a peak at 11 days post-challenge. The infection gradually stabilized at 40~80 days after infection. Subsequently, we started a dosing therapy with FLT (12.9/kg/day), T1144 (0.9/kg/day) and normal saline (as vehicle control) in this NHP model on day 85 after the challenge, followed by the programed dosing therapies as shown in Figure 7A and as described in the Materials and Methods Section. Blood was collected every 3 or 4 days to determine the plasma viral titer with a highly sensitive quantitative real-time RT-PCR (qRT-PCR) with the lower detection limit (LDL) of ~10^2^ RNA copies/mL normal saline. Before treatment, the viral load in rhesus monkeys was 4.22~5.89 (average 5.00) log10 RNA copies/mL.

As shown in Figure 7B, in the first stage of treatment (85~99 days post-challenge), the viral loads in the FLT and T1144 groups rapidly decreased to a low level, 2.45 and 2.27 log10 RNA copies/mL on average, respectively, while that in the normal saline group was still at a high level of 5.18 log10 RNA copies/mL on average. In the second stage (102~127 days post-challenge), viral loads in the FLT and T1144 groups remained at a low level of 2.71 and 3.31 log10 RNA copies/mL on average, respectively. In the third stage (130~172 days post-challenge), the average viral load in the FLT and T1144 groups was 2.98 and 3.93 log10 RNA copies/mL, respectively, while that of the normal saline group was 4.68 log10 RNA copies/mL on average. In the fourth stage (176~246 days post-challenge), the average viral load in the FLT group was 2.79 log10 RNA copies/mL, and viral load in about three-quarters of plasma samples was below the LOD, whereas the average viral load in the T1144 group had rebounded to 3.74 log10 RNA copies/mL. In the same period, the average viral load in the normal saline group was 4.91 log10 RNA copies/mL. 

## 3. Discussion

Albumin is the most commonly used natural protein for improving the half-life of biological drugs [26,27]. Its residence time in the blood is about 2 to 3 weeks, which is much longer than that of a synthetic polymer with the same molecular weight and hydrodynamic radius. Peptides or proteins can be directly linked to albumin through non-covalent binding [28,29]. The advantages of non-covalent bonding include good biocompatibility, tunable PK, easy production, and high stability, while the disadvantages include that only a few amino acid residues are feasible for modification, such as a single Cys and multiple Lys, and relatively low affinity for HSA. Also, the quality of each batch is difficult to control, and modification has resulted in higher costs [30]. However, an alternative method uses a small protein, or monobody, which contains ABD to mediate an indirect binding of a peptide or protein to albumin [31].

Monobody (antibody mimic) is a newly discovered synthetic binding protein with compact and thermally stable and independent variable structure [32,33,34]. This type of protein (5–20 kDa) has a smaller volume than antibody (~150 kDa), Fab or scFv (~25–50 kDa), and its variable region can be processed in vitro to produce a specific binding capacity not lower than the antibody [32]. The most widely used monobody is derived from the tenth human fibronectin type III domain (10Fn3). Its structure is similar to the complementarity determining region (CDR) of antibodies. It can be modified to form an artificial binding protein and bind to the target protein, such as albumin [35,36].

In this study, we constructed an ABD-containing monobody, FN3, to mediate the binding of T1144, a potent HIV fusion inhibitor, to HSA. FN3 has three (BC, DE and FG) loops at each end, and the positions of these loops correspond, approximately, to the positions of CDR1, 2 and 3 of the VH domain of IgG, respectively (Figure 2B). BC and FG loops are involved in the binding of FN3 to albumin. More importantly, FN3 does not have disulfide bonds; thus, it can be expressed in *E. coli* in large quantities with very high stability [37]. FN3 has small molecular size, good stability and obvious structural characteristics. It can be engineered to different targets based on FN3-binding proteins [37,38,39]. At the same time, the prokaryotic system can be used for mass production to avoid the key restriction factor of tedious and expensive production in the industrialization of mAb drugs. 

Here, we chose T1144, a potent HIV fusion inhibitor, as the coupling part since its C-terminal region contains the important pocket-binding domain, which makes the CHR-peptide more active and more stable. It also creates a high genetic barrier to resistance than that achieved with T20 [40]. T1144 has a half-life about 10-fold longer than that of T20 in cynomolgus monkeys. The half-life of T1144 is 12~15 h in humans, about 4-fold longer than that of T20 [41]. Based on the amino acid sequences of T1144, FN3 and L35 (a 35-mer linker) (Figure 2), we constructed plasmid FN3-L35-T1144-PHFT and used it to express the recombinant protein FLT. C10-T1144, which contains T1144, an L10 linker (Figure 1), and FN3, was included as a control. We found that FLT exhibited more potent inhibitory activity than C10-T1144 against infection of the laboratory-adapted HIV-1 strains (IIIB and Bal), while FN3 had no detectable antiviral activity (Figure 3). These results suggest that FN3 plays no role in the inhibition of HIV-1 infection and that the conjugation of FN3 to C35-T1144 does not affect the antiviral activity of a linker-linked T1144. 

More importantly, FLT inhibited infection by various clinical isolates, including A, B, C, D and AG groups with R5-tropism, X4-tropism or R5/X4 dual tropism, with an average IC_50_ of 14.8 nM, which is about 2.5-fold more potent than that of enfuvirtide (T20), which had an average IC_50_ of 51.6 nM (Table 1). Furthermore, FLT was more effective than enfuvirtide (T20) against T20-, NTI/NNRTI- and integrase-resistant strains (Table 2). With a mechanism of action similar to that of T20, FLT inhibited HIV-1 fusion with and entry into the host cells by interacting with the gp41 NHR-trimer and blocking 6-HB core formation between viral gp41 NHR and CHR domains. 

The key purpose of this study is to determine whether FLT has better stability and a longer half-life in vivo. Accordingly, we evaluated the half-life of FLT in rats through tail vein injection of FLT and found it to be 3.62- and 22.21-fold longer than that of T1144 and T20, respectively. It is predicted that FLT will stay longer in human body than in rats since the half-life of rat serum albumin in rats is about 1.9 days, while that of HSA in human blood circulation is 19 days [42,43]. The fusion of T1144 with a long flexible linker (L35) and an ABD-containing FN3 has resulted in significant enhancement of T1144’s anti-HIV-1 activity in vivo (Figure 6). Consequently, the potent and broad-spectrum nature of FLT indicates its suitability as a next-generation, long-acting HIV-1 fusion inhibitor for the treatment of patients resistant to other therapies.

Albuvirtide, a 3-maleimide acid (MPA)-modified T20-like HIV-1 fusion inhibitory peptide, was approved by the State Food and Drug Administration of China in 2018 for clinical use in combination with lopinavir/ritonavir (LPV/r) for intravenous infusion once a week [44]. Its long-acting effect stems from its irreversible binding to HSA, resulting in a prolonged half-life (t_1/2_ = 25.8 h in rats) [45]. FLT has similar half-life in rats (t_1/2_ = 27.1 h). If the FLT-based anti-HIV drug can be approved for clinical use in the future, it may also be used once a week. Since the binding of FLT to HSA is reversible, while binding of albuvirtide is non-reversible, FLT with smaller molecular size (22 kDa) is expected to be more accessible than HSA-bound albuvirtide (>70 kDa) to the constrained space of NHR-trimer in the fusion intermediate state [20]. Since FLT can be expressed in *E*. *coli* for large-scale production, the production cost of FLT protein is expected to be lower than that of a peptide drug. Ibalizumab (formerly TNX-355), as a mAb drug, can be administered once every two weeks. Its annual cost is more than 660,000 US dollars per year [46], while the cost of an FLT-based antiviral drug, if approved for clinical use, is expected to be lower than that of an antibody drug. Another long-acting HIV treatment drug is Cabenuva, which is a new combination consisting of cabotegravir, a new integrase inhibitor, and rilpivirine, an NNRTI [47]. However, before using this therapy, patients need to take one cabotegravir tablet and one rilpivirine tablet a day for one month to determine tolerability. The possibility of emergence of viral strains resistant to cabotegravir and/or rilpivirine in patients treated with this combination therapy for a long time cannot be excluded. The cost of this combination therapy is also high at ~48,000 USD per year. Therefore, FLT shows excellent promise for further development as a new long-acting anti-HIV drug for HIV-infected patients.

## 4. Materials and Methods

### 4.1. Materials

MT-2 cells, MT-4 cells, Laboratory-adapted, primary and drug-resistant HIV-1 strains, mouse anti-p24 antibody, and HIV immunoglobulin were obtained from the NIH HIV Reagent Program. Peptides N36, C34, C34-FAM, T20, T1144 and C10-T1144 were synthesized by a standard solid-phase 9- fluorenylmethyl carbonyl method using the Applied Biosystems model 433A peptide synthesizer. All peptides were purified to homogeneity (>95% purity) by high-performance liquid chromatography (HPLC) and identified by laser desorption mass spectrometry (PerSeptive Biosystems, Framingham, MA, USA). 

### 4.2. Construction of the Expression Vectors

The FN3-PHFT plasmid encoding a modified FN3 sequence was kindly provided by the Beijing Prosperous Biopharm Company. Briefly, the ABD fragment was inserted into the BC loop and FG loop regions of the PHFT vector. First, the FN3 sequence was amplified by PCR using plasmid FN3-PHFT as a template, and the L35-T1144 sequence was amplified from PGEX-6P-1MD1.1-L35-T1144 plasmid. Second, both fragments were digested with Bgl II and ligated. A flexible 35-mer linker consisting of GGGGSGGGGS was added between FN3 and T1144, allowing FN3 and T1144 in FLT to freely move around and interact with their targets. Then, the FN3-L35-T1144 DNA fragment was digested by BamH I and Xho I enzymes and linked to the PHFT plasmid and digested by the same enzymes to form a recombinant plasmid. The recombinant plasmid FN3-L35-T1144-PHFT was constructed, and its nuclear acid sequence was confirmed by direct DNA sequencing.

### 4.3. Expression and Purification of the Peptides

The expression strain *E. coli* BL21 (DE3) was transformed with FN3-L35-T1144-PHFT (FLT-PHFT) and Fn3-PHFT plasmids in the presence of kanamycin at 50 µg/mL. FLT-PHFT and Fn3-PHFT were induced at an optical density of 0.6 with 0.2 mM Isopropyl β- d-1-thiogalactopyranoside (IPTG) for 10 h at 16 °C. The protein was purified by using a Ni Sepharose column according to the manufacturer’s instructions (GE Life Sciences, Marlborough, MA, USA).

### 4.4. Inhibition of HIV-1 Infection

The inhibitory activities of FLT against laboratory-adapted viral strains HIV-1 IIIB (subtype B, X4) and Bal (subtype B, R5), the primary and drug-resistant HIV-1 strains were all detected as previously described [48]. Briefly, a series of two-fold dilutions (50 μL) of FLT were incubated with 100 TCID_50_ (50% tissue culture infective dose) of a viral strain for 30 min at 37 °C, and then 1 × 10^5^/mL MT-2 cells or M7 cells were added. The culture supernatants were changed after 12 hrs. Four or seven days later, the culture supernatants were collected for measurement of p24 antigen by ELISA as previously described [49]. The IC_50_ values were then calculated by using the CalcuSyn software [50] kindly provided by Dr. Chou at the Memorial Sloan-Kettering Cancer Center in New York.

### 4.5. Fluorescence Native Polyacrylamide Gel Electrophoresis (FN-PAGE)

FN-PAGE was used to determine the formation of 6-HB between the NHR- and CHR-peptides as previously described [40]. Briefly, each inhibitor to be tested was mixed with PBS, N36- or C34-FAM at a final concentration of 40 µM, incubated at 37 °C for 30 min, and mixed again with 1/5 volume of loading buffer (Tiandz, Beijing, China). The final mixture was loaded into 18% tris-glycine native gel at 20 μL per well, and gel electrophoresis was carried out with 125 V of constant voltage at room temperature for 2 h. The gel was imaged with a FluorChem 8800 imaging system and then stained with Coomassie Blue.

### 4.6. Detection of Inhibition of 6-HB Formation by ELISA

Inhibition of 6-HB formation was determined by ELISA as previously described [40]. Briefly, each well of a microplate was coated with 50 μL NC-1 MAb at 5 μg/mL in PBS buffer at 4 °C overnight and washed once with 200 μL ELISA washing buffer (PBS containing 0.1% Tween-20), followed by addition of 200 μL blocking buffer (0.5% gelatin in PBS) at 37 °C for 1 h. Then, 25 μL of an inhibitor to be tested at 20, 10, 5, 2.5, 1.25, 0.625 and 0.312 μM and 25 μL N36 in PBS, as well as 50 μL biotin-C34 in PBS (final concentration of N36 and biotin-C34 was 1 and 2 µM, respectively) were incubated at 37 °C for 1 h. Afterwards, 50 μL streptavidin-labeled HRP (1:1000, diluted with PBS) and TMB were added sequentially. The absorbance at 450 nm (A450) was measured using an ELISA reader (Ultra 386, Tecan, Durham, NC, USA).

### 4.7. Isothermal Titration Calorimetry (ITC)

The interaction affinity between the NHR-peptide and CHR-peptide was determined by ITC on a TA Instruments low volume Nano ITC calorimeter (TA Instruments, New Castle, DE, USA). The purified FLT protein and HSA were dialyzed in PBS solution at 4 °C for 16 hrs. Then, FLT (20 μM, 185 μL) and HSA (380 μM, 50 μL) were degassed with a vacuum pump (Thermovac) provided together with the Nano ITC calorimeter. FLT was added to the active cell, while HSA was placed in the titration needle. The sample volumes to the cell and titration were 200 μL and 50 μL. The titration was carried out at a constant temperature of 25 °C with a stirring rate of 250 rpm. The baseline balance level was intermediate. Each aliquot (2 μL per injection) was titrated into the cell for an automated sequence of 25 injections spaced at 160 s intervals. The data were analyzed using the TA-ITC software.

### 4.8. Pharmacokinetic Study

Sprague–Dawley (SD) rats were purchased from the Experimental Animal Center of Shanghai Medical College with an average body weight of 200 ± 10 g. Experiments on SD rats were approved by the Ethics Committee at Fudan University (Approval No. 2017-A046–01). Rats (*n* = 3) were given a single-dose injection with FLT, T1144 or T20 at the same concentration (5.94 mg/kg of FLT, 1.28 mg/kg of T1144 and 1.26 mg/kg of T20) intravenously (i.v.). The applied dose of FLT was chosen based on the results from our previous study [49]. Blood samples were collected from the orbital sinus at 0, 0.5, 1.5, 3, 6, 9, 12, 24, 48, 72, 96 and 120 h after injection of the inhibitors tested, respectively. The sera were isolated from the blood samples by centrifugation (3000 rpm/min) for 10 min at 4 °C and were stored at −80 °C until use. The determination of peptide and protein concentration was performed as described previously [51]. The PK parameters were calculated using the Non-Compartmental Analysis of Plasma Data software package (PKSolver 2.0). 

### 4.9. Rhesus Monkey Experiments

The NHP study was performed in accordance with the protocols (No. XJ17005) at the Institute of Laboratory Animal Science, Chinese Academy of Medical Sciences with the approval of Institutional Animal Care and Use Committee (IACUC). All animals were housed and cared for in a facility accredited by the American Association for the Assessment and Accreditation of Laboratory Animal Care (AAALAC) and the experimental procedures were performed in Animal Bio-Safety Level 3 (ABSL-3) laboratory. All monkeys were fed a commercially available monkey diet twice daily and supplemented with fresh fruit regularly. This study was conducted in the strict compliance to ensure safety and animal welfare, as previously described [52]. Rhesus monkeys (negative for simian immunodeficiency virus and simian T-lymphotropic virus) were randomly assigned into three groups (*n* = 4) for treatment with FLT, T1144 and saline, respectively. The dose of FLT applied to monkeys was estimated based on to dose of T20. They were inoculated intravenously with SHIV_SF162P3_ at 100 TCID_50_. FLT (12.9 mg/kg), and T1144 (0.9 mg/kg), or normal saline was subcutaneously injected daily starting at 85 days post-infection and then once every other day starting at 102 days, once every two days starting at 130 days, and once a week starting at 176 days until 246 days post-infection (Figure 7A). Plasma viral RNA loads were determined by a highly sensitive quantitative qRT-PCR as previously described [53]. The lower detection limit was approximately 10^2^ copies/mL. 

## 5. Conclusions

**Figure 8:**
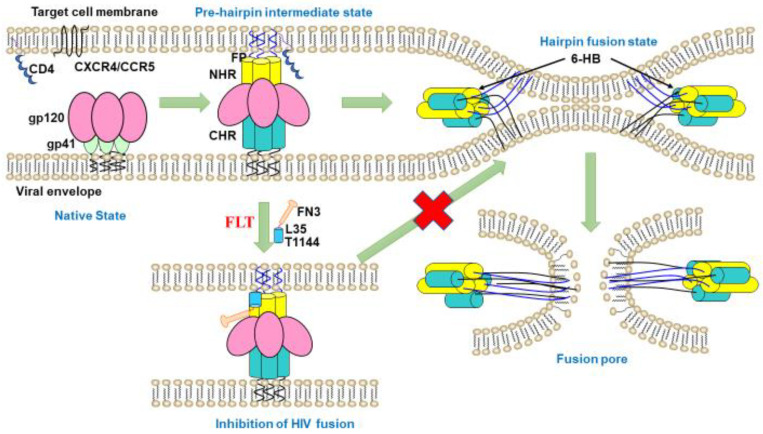
The mechanism of action of FLT in vitro. FLT binds to the HIV-1 gp41 NHR region, including the PFD, to block the formation of homologous 6-HB between viral NHR and CHR, thus inhibiting virus-cell membrane fusion.

**Figure 9:**
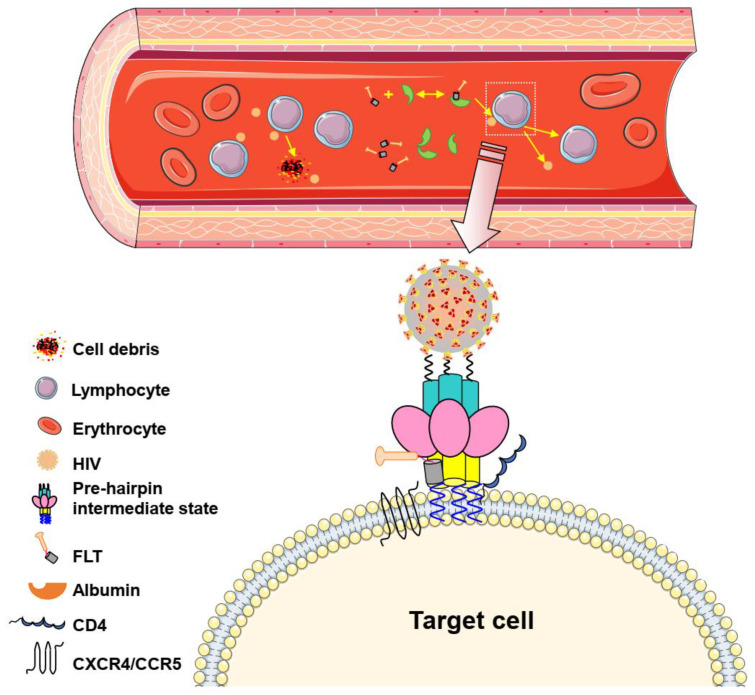
The mode of action of FLT in blood circulation. First, FLT binds to HSA reversibly. Then, FLT is gradually released into the blood to bind the HIV-1 gp41NHR region for block the formation of homologous 6-HB between the viral NHR and CHR, thus inhibiting virus-cell membrane fusion.

In summary, we have designed an HIV fusion inhibitor, termed FLT, which is linked with linker L35 to the long-acting protein FN3, which incorporates the specific albumin-binding domain (ABD) of HSA screened by phage display technology into the FN3 protein, thus obtaining specific binding capacity of albumin. FLT uses the extra-long half-life of HSA to extend the half-life of peptide drugs (Figure 8 and Figure 9). Subsequently, we evaluated the protective effect in the non-acute-stage rhesus monkey model and found that FLT effectively suppressed virus rebound for a long time. Particularly in the 4th stage when no FLT was given, the viral load in the SHIV-infected rhesus monkey model maintained at very low level (approaching to the detection limit), while the T1144 control group showed a rebound in viral load during this stage (Figure 7). Therefore, FLT is a promising candidate for the development of long-acting anti-HIV-1 agents.

## Figures and Tables

**Table 1:** Inhibitory activity of FN3, FLT, and enfuvirtide (T20) on infection of MT-4 cells by primary HIV-1 strains.

HIV-1 Clinical Isolates	IC_50_ (nM)		
	FN3	FLT	Enfuvirtide (T20)
HIV-1 96USSN20 (X4/R5, A)	>128	65.3 ± 2.6	55.3 ± 2.4
HIV-1 96USNG17 (X4, A)	>128	9.8 ± 0.9	49.3 ± 4.9
HIV-1 90US_873 (R5, B)	>128	6.5 ± 0.2	50.1 ± 2.0
HIV-1 BZ167 (X4, B)	>128	8.8 ± 0.3	53.9 ± 4.3
HIV-1 SE364 (R5, C)	>128	12.5 ± 0.5	45.2 ± 3.2
HIV-1 PBL288 (R5, C)	>128	9.3 ± 0.5	46.9 ± 2.1
HIV-1 92UG001 (X4/R5, D)	>128	11.7 ± 0.8	77.1 ± 6.8
HIV-1 J32228M4 (R5, D)	>128	6.4 ± 0.3	21.1 ± 1.1
HIV-1 DJ263 (R5, CRF02_AG)	>128	7.5 ± 0.3	55.6 ± 1.2
HIV-1 CAM1475MV (R5, CRF02_AG)	>128	10.2 ± 0.6	61.2 ± 1.3

Each sample was tested in triplicate and the experiment was repeated twice. The data from a representative experiment are presented as mean ± standard deviation.

**Table 2:** Inhibitory activity of FN3, FLT and T20 on infection by drug-resistant HIV-1 strains.

HIV-1 Drug-Resistant Strains	IC_50_ (nM)		
	FN3	FLT	Enfuvirtide (T20)
T20-resistant strains			
HIV NL4-3 (T20-sensitive strain)	>128	24.2 ± 2.0	20.8 ± 5.6
HIV NL4-3 N42T, N43K	>128	28.1 ± 1.8	>128
HIV NL4-3 V38E, N42S	>128	25.9 ± 1.0	>128
HIV NL4-3 V38A, N42T	>128	19.4 ± 1.5	>128
NRTI/NNRTI-resistant strains			
HIV-1 A018A (NRTI -sensitive)	>128	24.7 ± 1.7	37.9 ± 2.6
HIV-1 A012 (NRTI-resistant)	>128	28.6 ± 9.0	27.6 ± 5.1
HIV-1 IIIB A17 (NNRTI-resistant)	>128	23.2 ± 1.9	40.2 ± 4.6
Integrase inhibitor-resistant strains			
HIV-1 NL4-3 CA138763	>128	20.2 ± 0.6	31.2 ± 1.3
HIV-1 NL4-3 CA138764	>128	22.3 ± 2.1	35.3 ± 1.4
HIV-1 NL4-3 CA138767	>128	20.7 ± 0.3	34.6 ± 2.5

Each sample was tested in triplicate and the experiment was repeated twice. The data from a representative experiment are presented as mean ± standard deviation.

**Table 3:** Pharmacokinetics of FLT, T1144, and enfuvirtide (T20) in SD rats.

Peptide	T_1/2_ (h)	Tmax (h)	Cmax (μg/mL)	AUC (μg/mL/h)	CL (mL/h)
FLT	27.09 ± 6.9	0.5	863.2 ± 73.5	10624.1 ± 1583.2	0.063 ± 0.004
T1144	7.48 ± 0.4	0.5	147.4 ± 2.1	2338.9 ± 127.0	0.349 ± 0.019
T20	1.22 ± 0.2	1.5	305.0 ± 20.9	967.3 ± 83.4	0.855 ± 0.075

Each sample was tested in triplicate and the experiment was repeated twice. The data from a representative experiment are presented as mean ± standard deviation.

## Data Availability

Data is contained within the article and supplementary material.

## Supplementary Materials

The following supporting information can be downloaded at: https://www.mdpi.com/article/10.3390/ph15040424/s1, Figure S1: SDS-PAGE analysis of FN3 and FLT purified from 500 mL bacterial culture supernatant with Ni Sepharose column; Table S1. The identity and similarity of NHR and CHR sequences of the HIV-1 isolates tested.
