# Supplementary material for: A Protein-Based, Long-Acting HIV-1 Fusion Inhibitor with an Improved Pharmacokinetic Profile
Source: Pharmaceuticals (Basel). 2022 Mar 30;15(4):424. doi: 10.3390/ph15040424 (PMC9025429; doi:10.3390/ph15040424)
Supplement: Supplementary file 1 [file pharmaceuticals-15-00424-s001.zip › pharmaceuticals-1597366-supplementary.pdf]

Supplemental information

Table S1. The identity and similarity of NHR and CHR sequences of the HIV-1 isolates tested

**HIV-1 Isolate**	**NHR (548-614)**		**CHR (628-694)**	
	**Identity (%)**	**Similarity (%)**	**Identity (%)**	**Similarity (%)**
HIV-1 NL4-3 (X4, B)	100.0	100.0	100.0	100.0
HIV-1 96USSN20 (X4/R5, A)	NA	NA	73.1	89.6
HIV-1 96USNG17 (X4, A)	NA	NA	74.6	91.0
HIV-1 90US_873 (R5, B)	89.9	97.1	86.6	98.5
HIV-1 BZ167 (X4, B)	92.8	97.1	83.6	94.0
HIV-1 SE364 (R5, C)	NA	NA	80.6	91.0
HIV-1 PBL288 (R5, C)	87.0	97.1	73.1	85.1
HIV-1 92UG001 (X4/R5, D)	91.3	94.2	77.6	92.5
HIV-1 J32228M4 (R5, D)	92.8	95.7	85.1	92.5
HIV-1 DJ263 (R5, CRF02_AG)	88.4	95.7	70.0	91.0
HIV-1 CAM1475MV (R5, CRF02_AG)	88.4	97.1	73.1	88.1
**Average**	**90.1**	**96.3**	**77.7**	**91.3**

HIV-1 strain NL4-3 (GenBank: JQ975395.1). Its gp41 NHR sequence (aa 548-614) is shown below: STMGAASMTLTVQARQLLSDIVQQQNNLLRAIEAQQHLLQLTVWGIKQLQARILAVERYLK DQQLLGIW, and its gp41 CHR sequence (aa 628-694) is shown below: AVPWNASWSNKSLEQIWNNMTWMEWDREINNYTSLIHSLIEESQNQQEKNEQELLELDKW ASLWNWF.

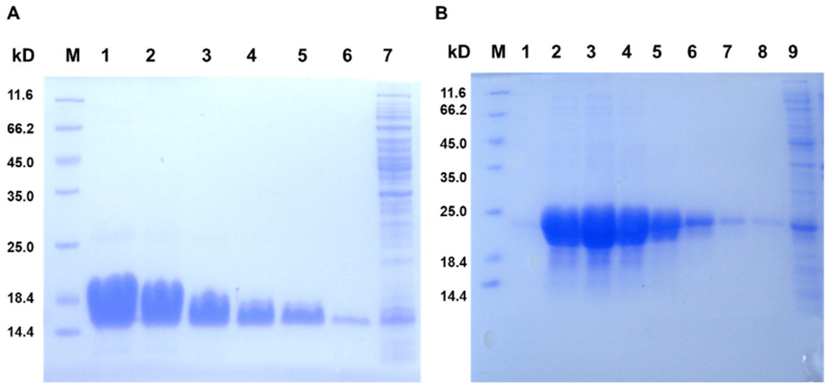

Figure S1. SDS-PAGE analysis of FN3 and FLT purified from 500 mL bacterial culture supernatant with Ni Sepharose column. (**A**) Purification of FN3. Lane 1-6: eluted fraction No. 1-6; lane 7: culture supernatant; (**B**) Purification of FLT. Lane 1-8: eluted fraction No. 1-8: lane 9: culture supernatant. Binding buffer: 20 mM sodium phosphate, 25 mM imidazole, and 500 mM NaCl, pH 7.4. Elution buffer: 20 mM sodium phosphate, 500 mM imidazole, and 500 mM NaCl, pH 7.
